# One Hour-Post-load Plasma Glucose ≥155 mg/dl in Healthy Glucose Normotolerant Subjects Is Associated With Subcortical Brain MRI Alterations and Impaired Cognition: A Pilot Study

**DOI:** 10.3389/fnagi.2021.608736

**Published:** 2021-02-04

**Authors:** Maria Perticone, Cherubino Di Lorenzo, Gennarina Arabia, Franco Arturi, Benedetto Caroleo, Bruno Tassone, Roberta Pujia, Teresa Vanessa Fiorentino, Carmelina Chiriaco, Giorgio Sesti, Aldo Quattrone, Francesco Perticone

**Affiliations:** ^1^Geriatrics Division, Department of Medical and Surgical Sciences, Magna Graecia University, Catanzaro, Italy; ^2^Department of Medico-Surgical Sciences and Biotechnologies, La Sapienza University Polo Pontino, Latina, Italy; ^3^Neurology Division, Department of Medical and Surgical Sciences, Magna Graecia University, Catanzaro, Italy; ^4^Internal Medicine Division, Department of Medical and Surgical Sciences, Magna Graecia University, Catanzaro, Italy; ^5^Geriatric Division, Azienda Ospedaliero-Universitaria Mater Domini, Catanzaro, Italy; ^6^Institute of Molecular Bioimaging and Physiology, National Research Council, Catanzaro, Italy; ^7^Department of Clinical and Molecular Medicine, La Sapienza University, Rome, Italy

**Keywords:** prediabetes, mild cognitive impairment, hippocampal volume, MRI, memory test

## Abstract

**Background:** Glucose alterations are associated with impaired cognition. The 1-h-post-load plasma glucose ≥155 mg/dl in non-diabetic subjects confers an increased risk of cardiovascular events and diabetes. This pilot study aimed to investigate whether the 1-h-post-load plasma glucose ≥155 mg/dl negatively affects the subcortical regions of the brain and the cognitive functions.

**Methods:** We enrolled 32 non-diabetic subjects. Patients were divided into two groups based on 1-h- post-load plasma glucose value > or < 155 mg/dl: normal glucose tolerance (NGT) 1-h-high and NGT 1-h-low subjects. All subjects underwent 3 Tesla MRI and standard neuropsychological tests.

**Results:** NGT 1-h-high subjects showed significantly lower values of both right (4.9 ± 0.9 vs. 5.1 ± 0.9 ml) and left (4.8 ± 1.1 vs. 5.1 ± 1.1 ml) hippocampal hemisphere volume, while right hemisphere hippocampal diffusivity was lower in the NGT 1-h-high group (10.0 ± 0.6 vs. 10.6 ± 0.5 10^−4^ mm^2^s^−1^). NGT 1-h-high subjects also showed a poorer memory performance. In particular, for both Rey Auditory Verbal Learning Task (RAVLT)—immediate-recall and Free and Cued Selective Reminding Test (FCSRT)—delayed total recall, we found lower cognitive test scores in the NGT-1 h-high group (26.5 ± 6.3 and 10.4 ± 0.3, respectively).

**Conclusions:** One-hour-post-load hyperglycemia is associated with morpho-functional subcortical brain alterations and poor memory performance tests.

## Introduction

Alterations of glucose metabolism, in particular impaired glucose tolerance (IGT) and type 2 diabetes mellitus (T2DM), have been associated with an increased incidence of Alzheimer's disease (AD) and vascular dementia (Exalto et al., 2012). Moreover, recently published data demonstrated a negative impact of chronically higher blood glucose levels on cognition, even in the absence of overt T2DM or IGT (Kerti et al., 2013). The association between diabetes and cognitive performance is known from many decades (Miles and Root, 1922), and over time, it has become evident that not only overt diabetes but also prediabetes are associated with cognitive alterations (Miles and Root, 1922; Carmichael et al., 2019). More recently, the Maastricht Study (Geijselaers et al., 2017) demonstrated a strong association between hyperglycemia and cognitive performance in diabetic patients independent of the cardiovascular (CV) context in which T2DM typically develops. Insulin resistance (IR) is a recognized factor in the appearance and progression of T2DM (Goldstein, 2002). Two different types of IR have been traditionally described: (1) a “*central*” IR, particularly expressed in the liver, and (2) a “*peripheral*” IR, particularly expressed in the muscles. Recently, the existence of a “*cerebral*” IR (Mielke et al., 2005) is defined as the failure of brain cells to respond to insulin and responsible for the appearance and progression of many forms of dementia and mild cognitive impairment (MCI) in late middle-aged adults (Willette et al., 2013, 2015). Insulin, insulin receptor, and its substrates are expressed in the whole central nervous system (Heni et al., 2014) where they regulate food intake and body weight, as well as regulate neurotransmitter release and synaptic plasticity (Arnold et al., 2018). A stream of human and experimental studies has provided convincing evidence that insulin may have an important role in many cognitive processes. Evidence indicates that the cognitive impairment commonly seen in many prediabetic/diabetic patients can be mediated by an altered signaling insulin-like growth factor (IGF) 1-insulin (Ye et al., 2002; Talbot et al., 2012). The brain structure that is particularly involved in diabetic-related MCI is the hippocampus (Convit et al., 2003; Biessels et al., 2008; Cherubin et al., 2012; Mortby et al., 2013). In particular, the measurement of mean diffusivity (MD) within the hippocampus represents an estimation of neuronal integrity, since it measures the overall degree of water diffusion in tissue and reflects the breakdown of cytoarchitecture and demyelinating processes. In addition to MD, fractional anisotropy (FA) measures the directional dominance of water diffusion and provides information about the density of nerve fibers (Beaulieu, 2002).

In recent years, we demonstrated that glucose normotolerant subjects who exhibit plasma glucose levels ≥155 mg/dl 1-h after an oral load of 75 g of glucose [normal glucose tolerance (NGT 1-h-high)] have a high prevalence of subclinical target organ damage; in particular, these subjects are characterized by an unfavorable CV risk profile (Succurro et al., 2009, 2010; Fiorentino et al., 2016) and are at an increased risk for future T2DM development (Fiorentino et al., 2015). This setting of subjects exhibits an insulin-resistant status characterized by enhanced insulin production but reduced tissue sensitivity to hormone action, and 1-h-post-load hyperglycemia can be regarded as an early marker of IR (Marcovecchio et al., 2017). Differently from prediabetic subjects, who show impaired fasting glucose (IFG) and/or IGT, NGT 1-h-high subjects have both fasting and 2-h-post-load normal plasma glucose levels.

Anyway, to our knowledge, no data are demonstrating a possible relationship between 1-h-post-load hyperglycemia and the cognitive status and hippocampal volume. Thus, we designed this pilot study to investigate if 1-h-post-load plasma glucose ≥155 mg/d negatively affects subcortical regions of the brain, in particular hippocampal volume and diffusivity and memory tests in a small cohort of glucose normotolerant subjects.

## Materials and Methods

For this observational cross-sectional pilot study, 32 consecutive healthy subjects (17 NGT 1-h-high and 15 NGT 1-h-low outpatients) were enrolled and referred to the Catanzaro University Hospital for the evaluation of their CV risk profile. The inclusion criteria contained: age ≥50 years, fasting plasma glucose <100 mg/dl, 2-h-post-load plasma glucose during the oral glucose tolerance test (OGTT) <140 mg/dl at visit 1, and the mini-mental state evaluation (MMSE) score = 30 at visit 1. The exclusion criteria contained: prediabetes (defined as IFG and/or IGT), T2DM, clinically evident dementia or cognitive impairment, previous diagnosis of any disease predisposing to the risk of cognitive impairment and/or the presence of any CV risk factor (i.e., dyslipidemia, hypertension, and obesity), liver cirrhosis, family history of dementia and/or cognitive impairment, history of alcohol or drugs abuse, the use of medications influencing glucose homeostasis or cognitive functions, contraindications to undergo cerebral 3 Tesla MRI, previous transient ischemic attack or stroke, and history of malignancies.

The local ethical committee approved the protocol, and informed written consent was obtained from all participants. All investigations were performed in accordance with the principles of the Declaration of Helsinki.

### Laboratory Determinations

All laboratory measurements were performed at visit 1 after a fast of at least 12 h. Plasma glucose was determined by the glucose oxidase method (Glucose Analyzer, Beckman Coulter S.p.A., Milan, Italy). Triglyceride and total low-density lipoprotein (LDL) and total high-density lipoprotein (HDL) cholesterol concentrations were measured by enzymatic methods (Roche Diagnostics GmbH, Mannheim, Germany). Serum insulin was determined in duplicate by a highly specific radioimmunoassay using two monoclonal antibodies; intra-assay CV 2.1% and inter-assay CV 2.9%. Insulin sensitivity was estimated by the Matsuda index/insulin sensitivity index (ISI), calculated as follows:

$$\begin{array}{l} {\text{ISI}}_{\text{MATSUDA}}\\ \text{=}\frac{10,000}{\sqrt{\;}{\text{(G}}_{\text{0}}{\text{[mmol/l] × I}}_{\text{0}}{\text{[mIU/l]) × (G}}_{\text{MEAN}}{\text{ [mmol/l] × I}}_{\text{MEAN}}\text{ [mIU/l])}}\text{,} \end{array}$$

(Matsuda and DeFronzo, 1999)

G_0_ = fasting plasma glucose concentration

I_0_ = fasting plasma insulin concentration

G_MEAN_ = mean plasma glucose concentration during the OGTT

I_MEAN_ = mean plasma insulin concentration during the OGTT

IR was estimated by homeostasis model assessment (HOMA-IR) according to the following equation:

HOMA = insulin (μU/ml) × glucose (mmol/L)/22.5 (Matthews et al., 1985).

A 75 g OGTT was performed with 0, 60, and 120 min sampling for plasma glucose and insulin levels measurements.

### Neuropsychological Assessment

To evaluate the presence of a cognitive impairment, a neuropsychological assessment for the measurement of cognitive functions was performed by the same expert-trained neuropsychologist (CC) at visit 2 (14 ± 3 days from visit 1), avoiding possible retroactive or proactive interferences. We used the following tests for the Italian population: Rey Auditory Verbal Learning Task (RAVLT)—immediate and delayed recall (Lezak et al., 2012), Rey–Osterrieth Complex Figure Test (ROCFT)—immediate and delayed recall (Lezak et al., 2012), and Free and Cued Selective Reminding Test (FCSRT) (Frasson et al., 2011). ROCFT scores were also used to assess the visuospatial constructional ability (Lezak et al., 2012).

### 3 Tesla Brain MRI

At visit 2, subjects have been examined using a MRI scanner Discovery MR750 3.0T (GE Healthcare, Chicago, IL, USA) with an eight-channel head coil. All participants underwent the same MRI protocol, including whole-brain 3D T1-weighted spoiled gradient recalled (SPGR) (BRAVO, voxel size of 1 × 1 × 1 mm^3^), conventional two-dimensional (2D) T2-weighted, and diffusion tensor imaging (DTI). A whole-brain T1-weighted scan was obtained in the sagittal plane (SPGR; echo time/repetition time (TE/TR) = 3.7/9.2 ms; flip angle 12°; voxel size 1 × 1 × 1 mm^3^); DTI data were constructed from a single-shot, diffusion-weighted spin echo-planar imaging sequence (TR = 8,000 ms, TE = 68.7 ms, field-of-view (FOV) = 21 × 21 cm^2^, matrix 96 × 64 (zero-padded to 256 × 256), slice thickness = 3.5 mm, 36 contiguous slices, applying parallel imaging with acceleration factor = 2; and acquired voxel size = 2.2 × 3.3 × 3.5 mm^3^, interpolated voxel size = 0.8 × 0.8 × 3.5 mm^3^). The maximum *b*-value was 1,000 s/mm^2^ in 25 non-collinear directions [number of excitations (NEX) = 1], and one volume was acquired without diffusion weighting (*b*-value = 0 s/mm^2^). The hippocampal structure was analyzed using the FSL v.5.0 software package. The hippocampal volume was extracted from the whole hippocampus through a previously published protocol (Cherubini et al., 2009), while the hippocampal microstructure was assessed by MD and FA using a DTI model fitted to each voxel. A full affine transformation between FA maps and brain-extracted whole-brain volumes from T1 was used to register DTI to the T1 image; then, the transformation matrix was applied to the MD maps. The T1 was registered to the FA map with an elastic registration algorithm. The hippocampal MD and FA were measured by computing the average MD and FA in the regions of interest (den Heijer et al., 2012).

### Statistical Analysis

Data were expressed as mean ± SD or as percent frequency, and comparisons between the groups were made by the *t*-test or the χ^2^-test, as appropriate. Differences were assumed to be significant at two-tailed values of *p* < 0.05. All analyses were performed using SPSS 20 for Mac.

## Results

Clinical, biochemical, and demographic characteristics of the whole study population and of the two groups separately are reported in Table 1. As expected, NGT 1-h-high subjects showed significantly higher 1-h-post-load plasma glucose values and lower IGF levels compared with NGT 1-h-low subjects. We did not find significant differences between the groups with regard to fasting and 2-h-post-load plasma insulin, while 1-h-post-load plasma insulin was significantly higher in the NGT 1-h-high group. Furthermore, we registered significant differences in both the Matsuda index and the HOMA values, with subjects in the NGT 1-h-high group showing an impaired insulin sensitivity compared with the NGT 1-h-low group.

**Table 1:** Clinical, biochemical, and demographic characteristics of the whole study population and of the two groups separately.

	**All *n* = 32**	**NGT 1-h-high**	**NGT 1-h-low**	***P*-value**
		***n* = 17**	***n* = 15**	
Age, years	60.4 ± 2.8	59.8 ± 3.3	61.3 ± 2.5	0.522
Sex, M/F	20/12	11/6	9/6	0.823
BMI, kg/m^2^	29.3 ± 1.1	29.0 ± 1.3	29.7 ± 0.5	0.408
Smoke, *n* (%)	4 (57)	2 (50)	2 (67)	0.683
SBP, mmHg	129.7 ± 18.1	122.0 ± 14.9	120.0 ± 19.3	0.219
DBP, mmHg	81.0 ± 7.8	77.6 ± 9.3	85.3 ± 1.1	0.230
Total Cholesterol, mg/dl	203.6 ± 44.8	171.7 ± 29.0	231.0 ± 16.0	0.134
HDL-cholesterol, mg/dl	51.6 ± 14.2	50.7 ± 15.2	52.7 ± 16.1	0.878
Triglycerides, mg/dl	125.4 ± 41.2	110.8 ± 37.7	145.0 ± 44.3	0.318
FPG, mg/dl	93.7 ± 4.7	92.7 ± 4.9	95.0 ± 5.3	0.824
1h plasma glucose, mg/dl	151.1 ± 35.7	174.0 ± 14.0	121.6 ± 33.2	0.035
2h plasma glucose, mg/dl	113.1 ± 23.6	108.5 ± 31.8	119.3 ± 7.4	0.595
Fasting insulin, UI/ml	14.1 ± 10.8	18.0 ± 13.5	9.0 ± 2.6	0.315
1 h insulin, UI/ml	127.5 ± 22.3	159.2 ± 34.6	79.3 ± 15.4	<0.0001
2 h insulin, UI/ml	81.3 ± 16.8	76.7 ± 12.9	94.5 ± 21.6	0.166
Matsuda index/ISI	2.9 ± 0.8	1.9 ± 0.5	4.4 ± 1.0	0.024
HOMA	3.3 ± 1.5	4.13 ± 1.1	2.1 ± 0.7	0.031
IGF-1, ng/ml	134.5 ± 28.3	114.4 ± 17.2	161.4 ± 7.7	0.007

*BMI, body mass index; DBP, diastolic blood pressure; FPG, fasting plasma glucose; HDL, high-density lipoprotein; HOMA, homeostasis model assessment; IGF, insulin-like growth factor; ISI, insulin sensitivity index; OGTT, oral glucose tolerance test; SBP, systolic blood pressure*.

In Table 2, we reported the 3 Tesla MRI parameters referred to as volume, anisotropy, and MD of all subcortical structures of the brain in the whole population and in the two groups separately. In comparison to NGT 1-h-low subjects, the NGT 1-h-high ones showed significantly lower values of left hemisphere cerebellum volume, of both left and right hippocampus volume, and of left hemisphere amygdala volume. On the contrary, in the same group, the MD of both the left and right hippocampus and MD of the left hemisphere caudate nucleus resulted higher with respect to the NGT 1-h-low group.

**Table 2:** 3 Tesla magnetic resonance parameters of the whole population and of the two groups separately.

	**All *n* = 32**	**NGT 1-h-high *n* = 17**	**NGT 1-h-low *n* = 15**	***P*-value**
Brain. Gray matter. Right Hemisphere. Volume, ml	379.4 ± 23.2	370.0 ± 24.1	393.4 ± 14.2	0.547
Brain. Gray matter. Right Hemisphere. Diffusivity, 10^−4^ mm^2^s^−1^	9.8 ± 0.6	10.0 ± 0.7	9.7 ± 0.4	0.345
Brain. Gray matter. Left Hemisphere. Volume, ml	382.8 ± 20.1	374.9 ± 22.7	394.7 ± 12.1	0.434
Brain. Gray matter. Left Hemisphere. Diffusivity, 10^−4^ mm^2^s^−1^	9.9 ± 0.6	10.1 ± 0.6	9.6 ± 0.4	0.444
Brain. White matter. Right Hemisphere. Volume, ml	342.2 ± 18.5	342.9 ± 21.4	341.2 ± 15.9	0.599
Brain. White matter. Right Hemisphere. Anisotropy	0.39 ± 0.08	0.40 ± 0.01	0.39 ± 0.01	0.767
Brain. White matter. Left Hemisphere. Volume, ml	347.8 ± 20.6	347.4 ± 24.1	348.4 ± 17.4	0.404
Brain. White matter. Left Hemisphere. Anisotropy	0.40 ± 0.08	0.40 ± 0.01	0.39 ± 0.01	0.062
Cerebellum. Gray matter. Right hemisphere. Volume, ml	55.8 ± 3.5	55.5 ± 2.9	56.4 ± 4.6	0.257
Cerebellum. Gray matter. Right hemisphere. Diffusivity, 10^−4^ mm^2^s^−1^	9.1 ± 0.6	9.1 ± 0.5	9.1 ± 0.7	0.735
Cerebellum. Gray matter. Left hemisphere. Volume, ml	56.2 ± 2.2	56.3 ± 1.4	56.4 ± 4.6	0.049
Cerebellum. Gray matter. Left hemisphere. Diffusivity, 10^−4^ mm^2^s^−1^	9.1 ± 0.6	9.0 ± 0.5	9.1 ± 0.7	0.551
Hippocampus. Right hemisphere. Volume, ml	5.0 ± 0.9	4.9 ± 0.9	5.1 ± 0.9	0.030
Hippocampus. Right hemisphere. Diffusivity, 10^−4^ mm^2^s^−1^	10.4 ± 0.61	10.0 ± 0.6	10.6 ± 0.5	0.022
Hippocampus. Left hemisphere. Volume, ml	4.8 ± 1.0	4.8 ± 1.1	5.1 ± 1.1	0.020
Hippocampus. Left hemisphere. Diffusivity, 10^−4^ mm^2^s^−1^	10.3 ± 0.9	10.3 ± 0.8	10.2 ± 1.1	0.051
Amygdala. Right hemisphere. Volume, ml	1.79 ± 0.16	1.77 ± 0.13	1.81 ± 0.23	0.064
Amygdala. Right hemisphere. Diffusivity, 10^−4^ mm^2^s^−1^	9.0 ± 0.3	9.1 ± 0.3	8.9 ± 0.3	0.421
Amygdala. Left hemisphere. Volume, ml	1.7 ± 0.2	1.7 ± 0.1	1.8 ± 0.3	0.047
Amygdala. Left hemisphere. Diffusivity, 10^−4^ mm^2^s^−1^	8.7 ± 0.5	8.7 ± 0.5	8.7 ± 0.7	0.417
Thalamus. Right hemisphere. Volume, ml	10.1 ± 0.9	9.8 ± 0.6	9.8 ± 0.5	0.148
Thalamus. Right hemisphere. Diffusivity, 10^−4^ mm^2^s^−1^	9.8 ± 0.5	9.8 ± 0.6	9.8 ± 0.5	0.611
Thalamus. Left hemisphere. Volume, ml	10.1 ± 1.2	10.0 ± 1.1	10.4 ± 1.4	0.877
Thalamus. Left hemisphere. Diffusivity, 10^−4^ mm^2^s^−1^	9.6 ± 0.7	9.7 ± 0.7	9.3 ± 0.7	0.906
Caudate Nucleus. Right hemisphere. Volume, ml	4.7 ± 0.4	4.6 ± 0.2	4.8 ± 0.6	0.119
Caudate Nucleus. Right hemisphere. Diffusivity, 10^−4^ mm^2^s^−1^	9.1 ± 0.9	9.4 ± 1.0	8.5 ± 0.6	0.323
Caudate Nucleus. Left hemisphere. Volume, ml	4.1 ± 0.4	4.0 ± 0.3	4.3 ± 0.4	0.569
Caudate Nucleus. Left hemisphere. Diffusivity, 10^−4^ mm^2^s^−1^	8.6 ± 1.0	8.9 ± 1.1	8.0 ± 0.4	0.033
Putamen. Right hemisphere. Volume, ml	6.2 ± 0.8	6.2 ± 1.0	6.2 ± 0.3	0.085
Putamen. Right hemisphere. Diffusivity, 10^−4^ mm^2^s^−1^	8.0 ± 0.2	8.1 ± 0.2	7.9 ± 0.1	0.226
Putamen. Left hemisphere. Volume, ml	6.5 ± 0.2	6.4 ± 0.5	6.7 ± 0.5	0.729
Putamen. Left hemisphere. Diffusivity, 10^−4^ mm^2^s^−1^	7.7 ± 0.2	7.7 ± 0.2	7.7 ± 0.2	0.674
Globus Pallidus. Right hemisphere. Volume, ml	7.4 ± 0.7	7.2 ± 0.2	7.7 ± 1.0	0.247
Globus Pallidus. Right hemisphere. Diffusivity, 10^−4^ mm^2^s^−1^	2.4 ± 0.3	2.3 ± 0.2	2.4 ± 0.4	0.227
Globus Pallidus. Left hemisphere. Volume, ml	7.9 ± 0.6	7.8 ± 0.4	7.7 ± 1.0	0.752
Globus Pallidus. Left hemisphere. Diffusivity, 10^−4^ mm^2^s^−1^	2.4 ± 0.2	2.3 ± 0.2	2.5 ± 0.2	0.051
Corpus Callosum. Splenium. Area, mm	145.0 ± 47.1	146.3 ± 46.4	143.0 ± 55.5	0.515
Corpus Callosum. Body. Area, mm	97.5 ± 30.5	95.2 ± 30.3	101.0 ± 35.2	0.507
Corpus Callosum. Genu. Area, mm	121.5 ± 43.4	116.4 ± 41.9	127.4 ± 47.7	0.565

Results of the memory performance tests are reported in Table 3. No differences between groups were detected with regard to the MMSE. The NGT 1-h-high group, in comparison to normal subjects, showed significantly a poorer performance in the following tests: immediate-recall and delayed-recall RAVLT, FCSRT immediate free recall, FCSRT delayed free recall, FCSRT delayed total recall, and ROCFT. Of note, for both the RAVLT immediate-recall and the FCSRT delayed total recall, we found pathological values in the NGT-1 h-high group.

**Table 3:** Memory performance test results in the whole study population and in the two groups separately.

	**All *n* = 32**	**NGT 1-h-high**	**NGT 1-h-low**	***P*-value**
		***n* = 17**	***n* = 15**	
MMSE	28.7 ± 1.2	28.2 ± 1.2	29.3 ± 1.1	0.290
RAVLT I.R.	37.1 ± 7.7	26.5 ± 6.3	45.1 ± 8.9	<0.0001
RAVLT D.R.	7.2 ± 2.3	4.8 ± 1.1	8.2 ± 2.0	<0.0001
FCSRT IFR	23.1 ± 3.3	21.5 ± 2.6	24.2 ± 3.6	0.024
FCSRT ITR	35.6 ± 0.5	35.0 ± 0.6	35.7 ± 0.6	0.950
FCSRT DFR	8.3 ± 1.7	8.7 ± 0.3	10.4 ± 2.5	<0.0001
FCSRT DTR	11.1 ± 0.9	10.4 ± 0.3	12.0 ± 1.0	0.006
FCSRT CSI	0.95 ± 0.05	0.95 ± 0.06	0.97 ± 0.05	0.877
ROCFT	29.1 ± 5.4	25.4 ± 4.5	31.1 ± 5.0	0.033

*CSI, cued sensitivity index; DFR, delayed free recall; DR, delayed recall; DTR, delayed total recall; FCSRT, Free and Cued Selective Reminding Test; IFR, immediate free recall; IR, immediate recall; ITR, immediate total recall; MMSE, Mini-Mental State Examination; RAVLT, Rey Auditory Verbal Learning Test; ROCFT, Rey–Osterrieth Complex Figure Test*.

## Discussion

The results of this study, even if very preliminary, demonstrate for the first time that healthy glucose normotolerant subjects with 1-h-post-load plasma glucose ≥155 mg/dl show a poorer memory function and visuoconstructive ability evaluated through ROCFT, together with a lower hippocampal volume and a higher hippocampal MD. In particular, this subset of patients showed a worse performance with regard to both immediate and delayed memory, as confirmed by the results of the neuropsychological tests.

The relationship between glucose homeostasis alterations and cognitive impairment/dementia is known for several decades. One possible explanation of the pathophysiological mechanisms underlying the appearance and progression of MCI in prediabetic/diabetic subjects is the mitochondrial overproduction of superoxide and the consequent activation of several pathways that are able to exert a toxic effect on the brain due to intracellular hyperglycemia. Glucose toxicity on neuronal structures may be exerted through a generation/scavenging imbalance of reactive oxygen species or through the advanced glycation of structural proteins in the brain (Geijselaers et al., 2017). Anyway, the hypothesis of a direct effect of hyperglycemia on the cognitive function of diabetic patients is quite controversial, since some evidence demonstrated that hyperglycemia *per se* is only weakly associated with cognitive performance (Brownlee, 2001). Furthermore, the study by Kerti et al. (2013) demonstrated a significant association between both long-term (HbA1c) and short-term (fasting plasma glucose) markers of hyperglycemia and worse cognitive performance and lower hippocampal volume in healthy subjects. Similar results have been obtained in the present study, in which 1-h-post-load hyperglycemia was associated with a lower hippocampal volume and a higher MD. These findings are in line with previously published results obtained in patients with T2DM (Biessels et al., 2008; Geijselaers et al., 2015), in patients with IGT (Convit et al., 2003), in patients with IFG (Cherubin et al., 2012), or in healthy subjects (Willette et al., 2013). A possible explanation could be found in the increased inflammatory response subsequent to hyperglycemia and the activation of the coagulation cascade, leading to subclinical strokes and, in turn, volume loss (Yaffe et al., 2004; Kale et al., 2006). Moreover, direct glucose toxicity may impair the integrity of the neuronal membrane, leading to an increase in extracellular water content and, as a consequence, an increased MD of the cerebral structures (Pocai et al., 2005).

Another important finding emerged in the present study is that NGT 1-h-high subjects showed higher values of HOMA, an indirect measure of IR. This condition is a well-recognized pathophysiological mechanism underlying the appearance and progression of T2DM and all glucose metabolism alterations; in the last decades, cerebral IR has been considered one of the central features of several forms of dementia, even in the absence of diabetes (Willette et al., 2013, 2015). Insulin receptors are expressed in several brain structures, especially the hypothalamus and the hippocampus. In particular, insulin action on the hypothalamus exerts a regulatory effect on metabolic pathways in the liver (Pocai et al., 2005) and in the adipose tissue (Scherer et al., 2011), while an altered hormone action at the hippocampal level may be responsible for cognitive alterations (Zhang et al., 2015).

In conclusion, the results of this pilot study, if confirmed in a wider population, could expand present information about the comprehension of the complex pathophysiological mechanisms underlying the appearance of cognitive disorders in subjects with very early glucose metabolism alterations. Furthermore, a piece of deeper knowledge about the possible implications of 1-h-post-load hyperglycemia could lead to the definition of this alteration as a “pre-prediabetic” status.

The present study has several limitations. First, this is a pilot study, in which the small sample size and the study design do not allow researchers to reach a definitive conclusion about the pathophysiological mechanisms underlying the appearance of MCI in this setting of subjects. Further studies with a larger sample size and a longitudinal observation are needed to confirm our hypothesis. Furthermore, we only tested the memory domain with standard neuropsychological tests, but we do not have information about the other domains that need to be tested in a wider population. Finally, the control group consists of “very healthy” subjects, probably not so representative of the general population.

## Data Availability Statement

The raw data supporting the conclusions of this article will be made available by the authors, without undue reservation.

## Ethics Statement

The studies involving human participants were reviewed and approved by Comitato Etico Calabria Centro. The patients/participants provided their written informed consent to participate in this study.

## Author Contributions

MP, GA, and FA: conceptualization. CDL: methodology. CC: software. GS, AQ, and FP: validation. MP: formal analysis. BC, TF, BT, and RP: investigation. MP and CDL: writing—original draft preparation. GA and FA: writing—review and editing. MP and FP: supervision. All authors have read and agreed to the published version of the manuscript.

## Conflict of Interest

The authors declare that the research was conducted in the absence of any commercial or financial relationships that could be construed as a potential conflict of interest.
